# Tip110 binding to U6 small nuclear RNA and its participation in pre-mRNA splicing

**DOI:** 10.1186/s13578-015-0032-z

**Published:** 2015-07-23

**Authors:** Ying Liu, Jinfeng Liu, Zenyuan Wang, Johnny J He

**Affiliations:** Department of Cell Biology and Immunology, Graduate School of Biomedical Sciences, University of North Texas Health Science Center, 3500 Camp Bowie Blvd., Fort Worth, TX 76107 USA; Department of Infectious Diseases, The First Affiliated Hospital of Medical College, Xi’an Jiaotong University, Xi’an, 710061 Shaanxi China; Department of Forensic Science, College of Medicine, Xi’an Jiaotong University, Xi’an, 710061 Shaanxi China

**Keywords:** Tip110, Small nuclear RNA, U6, RNA binding, Pre-mRNA, Splicing

## Abstract

**Background:**

RNA–protein interactions play important roles in gene expression control. These interactions are mediated by several recurring RNA-binding motifs including a well-known and characterized ribonucleoprotein motif or so-called RNA recognition motif (RRM).

**Results:**

In the current study, we set out to identify the RNA ligand(s) of a RRM-containing protein Tip110, also known as p110^nrb^, SART3, or p110, using a RNA-based yeast three-hybrid cloning strategy. Six putative RNA targets were isolated and found to contain a consensus sequence that was identical to nucleotides 34–46 of U6 small nuclear RNA. Tip110 binding to U6 was confirmed to be specific and RRM-dependent in an electrophoretic mobility shift assay. Both in vitro pre-mRNA splicing assay and in vivo splicing-dependent reporter gene assay showed that the pre-mRNA splicing was correlated with Tip110 expression. Moreover, Tip110 was found in the spliceosomes containing pre-spliced pre-mRNA and spliced mRNA products. Nonetheless, the RRM-deleted mutant (ΔRRM) that did not bind to U6 showed promotion in vitro pre-mRNA splicing, whereas the nuclear localization signal (NLS)-deleted mutant ΔNLS that bound to U6 promoted the pre-mRNA splicing both in vitro and in vivo. Lastly, RNA-Seq analysis confirmed that Tip110 regulated a number of gene pre-mRNA splicing including several splicing factors.

**Conclusions:**

Taken together, these results demonstrate that Tip110 is directly involved in constitutive eukaryotic pre-mRNA splicing, likely through its binding to U6 and regulation of other splicing factors, and provide further evidence to support the global roles of Tip110 in regulation of host gene expression.

## Background

In eukaryotic cells, gene expression begins in nucleus with transcription of protein-coding genes to primary RNA transcripts, or so-called pre-messenger RNA (pre-mRNA) or heterogeneous nuclear RNA (hnRNA) [1]. The pre-mRNA are processed to become mature mRNA through a series of post-transcriptional modifications [2]. One of them is the precise excision of noncoding sequences (introns) from pre-mRNA in the spliceosomes [2]. The spliceosomes are formed through assembly of spliceosomal small nuclear ribonucleoprotein (snRNP) and recruitment of numerous splicing factors to pre-mRNA [1, 2]. Efficient and proper pre-mRNA splicing is a critical step in gene expression control [3, 4]. Dysregulation of splicing elements or splicing regulators and subsequent abnormal pre-mRNA splicing result in expression of aberrant protein products and development of diseases [3, 4]. For example, overexpression of splicing factor SF2/ASF has been shown to trigger malignant transformation [5].

snRNP are important components of the spliceosomes in which the pre-mRNA splicing occurs [6]. There are five major snRNP U1, U2, U4, U5, and U6 in the spliceosomes containing small nuclear RNA (snRNA) U1 snRNA, U2 snRNA, U4 snRNA, U5 snRNA, and U6 snRNA, respectively [6]. Those snRNA are transcribed in the nucleus and exported to the cytoplasm after acquiring a m7G-cap [1, 2]. The core snRNP are initially assembled in the cytoplasm to contain snRNA, survival motor neuron protein, Gem-associated proteins, and Sm proteins, followed by nuclear import [7]. Once the core snRNP get into the nucleus, they enter the Cajal body, undergo further modifications such as RNA-targeted ribose methylation and pseudouridylation, recruit additional proteins to complete its final assembly for pre-mRNA splicing [7]. One group of the best-defined proteins that snRNP recruit in the Cajal body are serine-/arginine-rich repeat (RS domain)-containing splicing factors called SR proteins [8]. SR proteins are characterized by the presence of RNA recognition motifs (RRM) in the N terminus and the presence of RS domain in the C terminus. Most of the serine/arginine-rich (SR) proteins are found in the nucleus, specifically in nuclear speckles, and are known for their roles in alternative and constitutive pre-mRNA splicing [9, 10]. Besides SR proteins, there are a number of other pre-mRNA splicing factors in the nuclear speckles. Those pre-mRNA splicing factors are constantly moving between nuclear speckles and the nucleoplasm or other nuclear substructures [11]. Nevertheless, nuclear speckles are often regarded as the sites for storage, assembly, and modification of the splicing factors as well as the supply sources of splicing factors for active transcription sites [12–14]. In addition, nuclear speckles have been shown to be directly involved in the pre-mRNA splicing [15].

Tip110, also known as p110^nrb^, SART3, or p110, has been proposed to serve several important biological functions. These include tumor antigen function [16–18], control of gene transcription [19–21], regulation of protein stability [22, 23], and stem cell pluripotency and differentiation [24–26]. In addition, Tip110 has been proposed to be directly involved in pre-mRNA splicing through interaction with RNPS1 [16], U4/U6 snRNP [27, 28], YB-1 [29], and C-MYC [26]. In agreement with Tip110 roles in pre-mRNA splicing, Tip110 has two RNA recognition domains [16] and has been detected in the nuclear substructures Cajal bodies and nuclear speckles [14, 30, 31]. Further supporting Tip110 roles in pre-mRNA splicing is the recent findings that Tip110 directly binds to unphosphorylated C-terminal domain (CTD) of RNA polymerase II in the direct manner [21]. In the current study, we set out to identify specific RNA targets, if any, that might be involved in interaction with Tip110 and account for Tip110 function in pre-mRNA splicing. We took advantage of the RNA–protein binding-based yeast three hybrid screening strategy and identified snRNA U6 from a hybrid RNA library as putative Tip110-binding partner. We then characterized the interaction and its role in pre-mRNA splicing.

## Methods

### Cell lines and cell transfection

293T and Hela cells were purchased from American Tissue Culture Collection (ATCC, Manassas, VA, USA) and maintained in Dulbecco’s modified Eagle’s medium supplemented with 10% fetal bovine serum, in a 37°C, 5% CO_2_ incubator. Cells were transfected using the standard calcium phosphate precipitation method.

### Plasmids

Plasmids pTip110.His, pTip110s.His, and deletion mutants ∆NLS and ∆RRM, and pGEX-Tip110, pGEX-Tip110s, and deletion mutants ∆NLS and ∆RRM, and pAD.Tip110 were described elsewhere [20]. The standard PCR cloning technique was used to construct pTip110∆LSM, with pTip110.His as the templates and primers 5′-GGA ATT CAC CAT GGC GAC TGC GGC CGA A-3′ and 5′-CCG CTC GAG TCA ATG ATG ATG ATG ATG ATG GGC AAC TGC AGG AGC CG-3′ (the restriction enzyme sites underlined). The Tip110 siRNA expression plasmid was constructed in the backbone of pSHAG-1 by annealing oligonucleotides 5′-CG*G*GAT CCG ACT CAG CCT CGG GTT CTG AA-3′ and 5′-CGG GAT CCA AAA AAT TGG ACT CAG CCT CA-3′, and inserting the annealed DNA at the Bam HI site of pSHAG-1. All recombinant plasmids and deletion mutants were verified by sequencing. The sources of the other plasmids used in the study are: pGEX-4T-3 from Amersham (Piscataway, NJ, USA), pSHAG-1 from Dr. Gregory Hannon, pIIIA.MS2 from Dr. Marvin Wickens, pT7.U6 from Dr. Iain Mattaji, pDM138 from Dr. Thomas Hope, pcRev from Dr. Bryan Cullen, and pSP64-Hβδ6 from Dr. Adrian Krainer.

### Construction of a hybrid RNA library and yeast three-hybrid screening

A hybrid RNA library and yeast three-hybrid screening were performed as previously described [32], with some modifications. Briefly, genomic DNA was isolated from 293T cells and digested by Mse I, Tsp509I, Alu I and Rsa I, and then by T4 DNA polymerase in the presence of 100 μM dNTP. Digested DNA was fractionated on a 2% agarose gel to obtain DNA fragments of 50–150 bp. These small DNA fragments were then ligated into SmaI-linearized and calf intestine phosphatase-treated pIIIA.MS2 plasmid. The ligation mixture was transformed into GC5 competent bacteria for propagation and isolation of the library DNA. Yeast strain L40coat stably expressing LexA-MS2 coat fusion protein was first transformed with pAD-Tip110 plasmid and then with the RNA expression library. The selection of transformed yeast was performed on synthetic complete plates containing 0.5 mM 3-aminotriazole and no tryptophan, leucine, uracil and histidine, followed by X-gal filter assay for β-galactosidase expression. β-galactosidase-positive colonies were further cultured to isolate the hybrid RNA plasmids.

### Recombinant protein purification

GST-Tip110, GST-Tip110s, Tip110 deletion mutants ∆NLS and ∆RRM, and GST proteins were expressed in and purified from *E. coli* BL21, as previously described [20]. Briefly, expression plasmids were transformed into BL21 and induced with 1 mM isopropyl-d-thiogalactopyranoside for 2 h at 37°C. GST fusion proteins were purified using a Pierce GST fusion protein purification kit (Rockford, IL, USA). When necessary, GST was removed by treating the eluted protein with thrombin protease (10 units) (Invitrogen, Carlsbad, CA, USA) at room temperature for 18 h. The digested protein solution was dialyzed overnight in 4 l of phosphate-buffered saline and cleared of GST protein by additional incubations with fresh glutathione beads. The purified proteins were electrophoresed on a 8% SDS-polyacrylamide gel and stained with Gold–Blue (Pierce) to ensure the complete removal of GST protein as well as undigested fusion protein and other contaminated proteins.

### RNA–protein gel mobility shift assay

U6 RNA was synthesized using an Ambion in vitro transcription kit (Austin, TX, USA). Briefly, pT7-U6 plasmid was linearized with Dra I and incubated with ATP, CTP, GTP and ^32^P-labeled UTP in the presence of T7 RNA polymerase at 30°C for 1 h, followed by removal of free nucleotides by an CentriSpin column (Princeton Separation, Princeton, NJ, USA). ^32^P-labeled-U6 RNA was then mixed with purified GST-Tip110 or its mutant proteins in a total volume of 20 μl containing 1 mM HEPES, pH 7.6, 1 μM MgCl_2_, 16 mM KCl, 2.5% glycerol (v/v), and 10 nM DTT. The mixture was incubated on ice for 15 min and then subjected to 5% nondenaturing polyacrylamide gel electrophoresis (acrylamide-bisacrylamide, 80:1) in the 0.5× TBE buffer. The gel was dried and exposed for autoradiography.

### Preparation of pre-mRNA and in vitro splicing assay

Human β-globin minigene pre-mRNA was synthesized using an Ambion in vitro capped RNA transcription kit (Ambion). Briefly, pSP64-Hßδ6 plasmid was linearized with BamH I and incubated with ATP, CTP, GTP, cap anolog and ^32^P-labeled UTP at 37°C for 2 h. ^32^P-labeled pre-mRNA was then purified on a 5% denaturing polyacrylamide gel and suspended in 10 mM Tris.HCl, pH 7.9. The ^32^P-labeled pre-mRNA was then aliquoted and stored at −80°C. Splicing reactions were carried out in a 25 μl volume containing 12.8 μM MgCl_2_, 500 μM ATP, 20 mM creatine phosphate, 2.7% (w/v) polyvinyl alcohol, 1,000 U/ml RNasin, 12.8 mM HEPES, pH 8.0, 14% (v/v) glycerol, 62 mM KCl, 0.12 mM EDTA, and 0.7 mM DTT at 30°C for 2 h. The reactions contained 6 μl Hela nuclear extract (Promega, Madison, WI, USA) and 12.5 ng ^32^P-labeled pre-mRNA prepared above.

### Monoclonal antibody production

Anti-Tip110 monoclonal antibodies were produced by Promab Biotechnologies, Inc. (Albany, CA, USA). Briefly, purified GST-Tip110 protein was used to immunize BALB/c mice. The mice were given an additional boost immunization after 3 weeks. Three days after the boost, the spleen cells were isolated from the immunized mice and fused with SP20 cells (ATCC) using polyethylene glycol (Sigma, St. Louis, MO, USA). Resulting hybridoma were screened to be specific anti-Tip110 antibody using ELISA. To purify the monoclonal antibody, the hybridoma cells were cultured in a production module of the miniPERM device (Viva Science, Inc.), and the antibody was further purified from the culture supernatant using a Protein A column and a AKTA prime-automated liquid chromatography system (Amersham, Piscataway, NJ, USA). The antibody protein purity was determined to be IgG_2_ with a purity of higher than 99%.

### Tip110 immunodepletion

Purified Tip110 monoclonal antibody (20 μg) was incubated with 50 μl of 50% slurry protein A-Sepharose (Amersham) in a buffer containing 20 mM HEPES, pH 8.0, 100 mM KCl, 0.2 mM EDTA, 20% (v/v) glycerol, 0.5 mM PMSF, 1 mM DTT at 4°C for 4 h with continuous mixing. The protein A beads were then recovered by centrifugation and repeated washes with the same incubation buffer to remove excess anti-Tip110 antibody. An aliquot of the beads were then incubated with Hela nuclear extract at 4°C for 2 h with continuous mixing, and the supernatant was used as Tip110-depleted Hela nuclear extract. Tip110 protein on the immunoabsorbed beads was eluted by adding 20 μl of 4× SDS sample buffer, followed by boiling 5 min. The eluent and aliquot of the Tip110-depleted Hela nuclear extract were subjected to 10% SDS-PAGE gel and Western blot analysis to ensure efficient Tip110 depletion.

### Pre-mRNA splicing-dependent CAT reporter gene assay

293T cells were plated in a 6-well plate at a density of 0.72 × 10^6^ cells/well and 1 day later transfected with 1 μg pDM138, 1 μg pcRev, 1–5 μg pTip110.His or pTip110.siRNA. In all transfections, a CMVβGal plasmid was included to normalize transfection variations, and pcDNA3 was used to equalize the amount of transfected DNA. Forty-eight hours after transfection, the cells were harvested and subjected to 3 rounds of freeze–thaw in 0.25 M Tris.HCl, pH 8.0, and the lysates were collected for β-galactosidase and CAT activities. β-galactosidase was determined using a colorimtric assay [20], while CAT was determined by one-step simple phase extraction and scintillation counting as described [33].

### Preparation of cell lysates and Western blot analysis

Cells were washed twice with ice-cold phosphate-buffered saline (PBS), and cell pellets were suspended in two volumes of whole cell lysis buffer containing10 mM NaHPO_4_, 150 mM NaCl, 1% Triton X-100, 0.1% SDS, 0.2% sodium azide, 0.5% sodium deoxycholate, 0.004% sodium fluoride, and 1 mM sodium orthovanadate and incubated on ice for 10 min. Cell lysates were obtained by centrifugation, removal of the cell debris, and electrophoretically separated on 10% SDS-PAGE and analyzed by immunoblotting. Anti-His antibody was from Qiagen (Valencia, CA, USA), while β-actin was from Santa Cruz Biotech. (Santa Cruz, CA, USA). The blots were first probed with primary antibodies, followed by appropriate horseradish peroxidase-conjugated secondary antibodies, and then visualized with the ECL system (Amersham).

### RNA isolation and RNA-Seq

Hela were transfected with human Tip110 siRNA or the universal control siRNA. Total RNA was purified from transfected cells using Trizol according to the manufacturer’s instructions (Life Technologies, Grand Island, NY, USA) and used for RNA-Seq. The RNA-Seq was performed at the Genomic Core of UT Southwestern Medical Center, Dallas, TX, USA. The RNA-Seq reads were aligned to the human genome sequences for further differential and spliceosome pathway analysis using the Panther Pathway Analysis software.

### qRT-PCR

Total RNA was extracted using Trizol and used to synthesize cDNA using a ScriptII RT reagent kit (Promega). cDNAs were used for qPCR using a Power Sybr^®^Green PCR kit (Life Technologies) according to the manufacturer’s instructions. The qPCR program consisted of one cycle of 95°C for 10 min, 40 cycles of 95°C for 15 s and 60°C for 1 min.

## Results

### Identification of Tip110 RNA ligands by the yeast three-hybrid cloning

**Figure 1:**
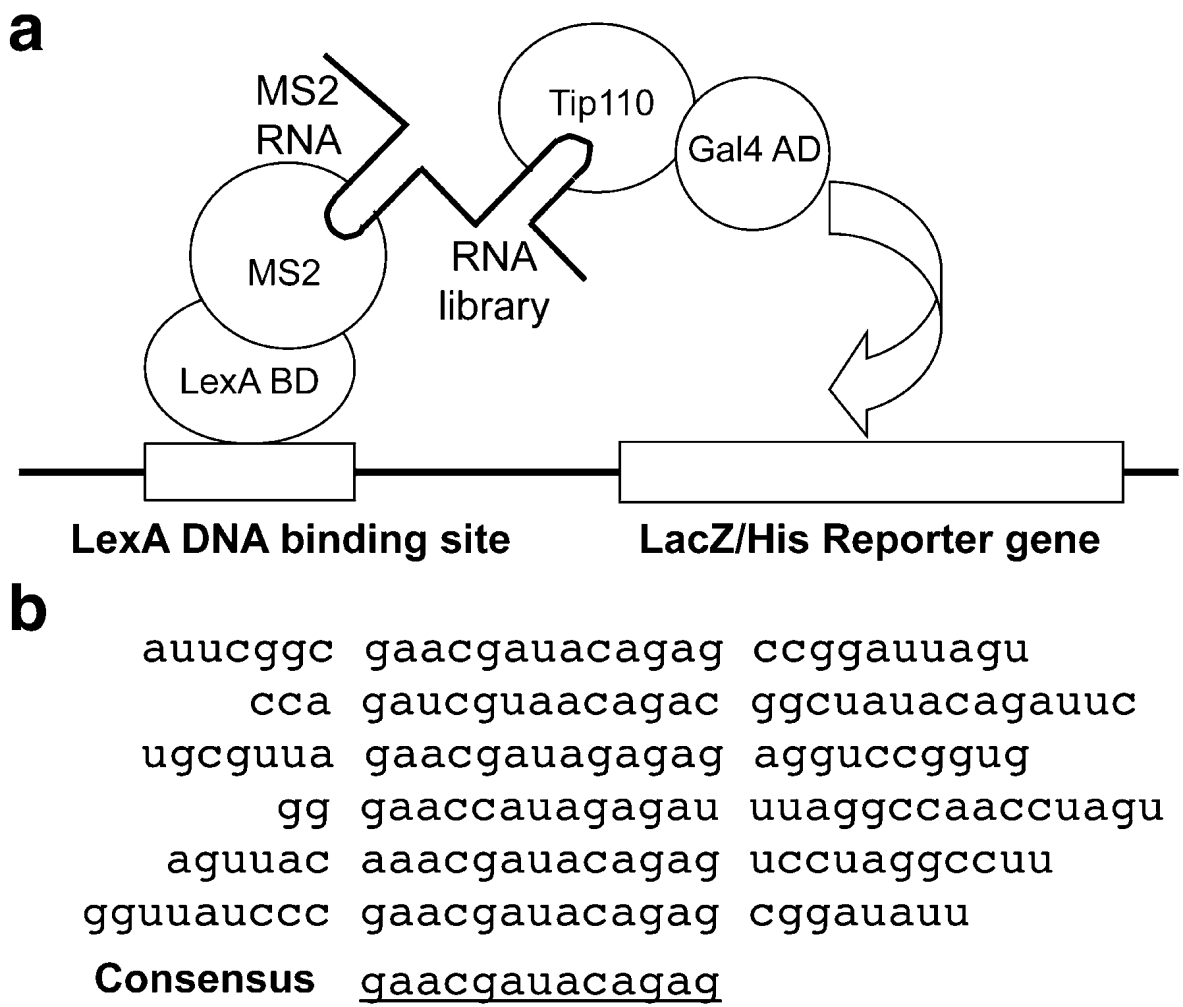
Identification of U6 as a Tip110 binding RNA target. **a** Scheme of the yeast three-hybrid cloning strategy. LexA BD-MS2: LexA DNA binding domain fused with phage coat protein MS2; MS2 RNA–RNA library: the target RNA sequence of MS2 protein was fused to a pool of RNAs transcribed from human genomic DNA; Gal4 AD-Tip110: Gal4 activation domain fused to Tip110 protein. Respective binding of MS2 protein to MS2 target RNA and Tip110 protein to its putative RNAs leads to activation of LacZ and His reporter genes. **b** Sequences of RNA hits obtained from the yeast three-hybrid screening using Tip110 as the bait. The consensus sequence was matched to small nuclear U6 RNA at nucleotides 34–46.

To determine if Tip110 bound to specific RNAs, we took advantage of a RNA–protein interaction-based yeast three-hybrid cloning strategy [34]. This genetic assay was initially developed to study RNA–protein interactions and later adapted to identify RNA ligands of an RNA binding protein [32]. It is based on the transcriptional activation of separable domains of transcriptional factors such as GAL4 and LexA, and is composed of two hybrid proteins and one hybrid RNA (Figure 1a). One hybrid protein consists of the DNA binding domain of transcriptional activator LexA fused to a known RNA binding protein bacteriophage MS2 coat protein. The hybrid RNA is a RNA library expressing a MS2 coat protein RNA binding sequence. The other hybrid protein consists of the activation domain of transcriptional activator Gal4 fused to a bait protein, in our case Tip110. Binding of the bait protein Tip110 to the target RNA in the RNA hybrid brings closer the Gal4 activation domain and the LexA DNA binding domain and subsequently activates expression of the reporter genes LacZ/His. We constructed a hybrid RNA library from 50 to 150 bp fragments of genomic DNA isolated from 293T cells and transformed it into the L40coat strain containing both LexA-MS2 and Gal4 AD-Tip110. We then plated the transformants on synthetic media lacking uracil, leucine, tryptophan, and histidine, which are used to select for the presence of these three hybrids and activation of the His reporter gene. We also include 0.5 mM 3-aminotriazole to reduce a background level of His gene expression. Among an estimate of 11.4 million yeast transformants, about 450 colonies were formed after 1 week. Of these, 156 were found to be β-galactosidase-positive. We then cultured these colonies in rich media to lose the Gal4 AD-Tip110 plasmid and tested them for their ability to activate reporter genes. The results showed that of these 156 colonies only 6 were no longer β-galactosidase-positive, while other 150 appeared to activate LacZ gene expression in the absence of Gal4 AD-Tip110. Sequence analysis of these RNA inserts revealed a consensus of 13 nucleotides (nt.): gaacgauacagag (Figure 1b). We performed a BLAST search with the sequence, and found it to be identical to small nuclear RNA U6 at nt. 34–46.

### Binding of Tip110 protein to U6 RNA

**Figure 2:**
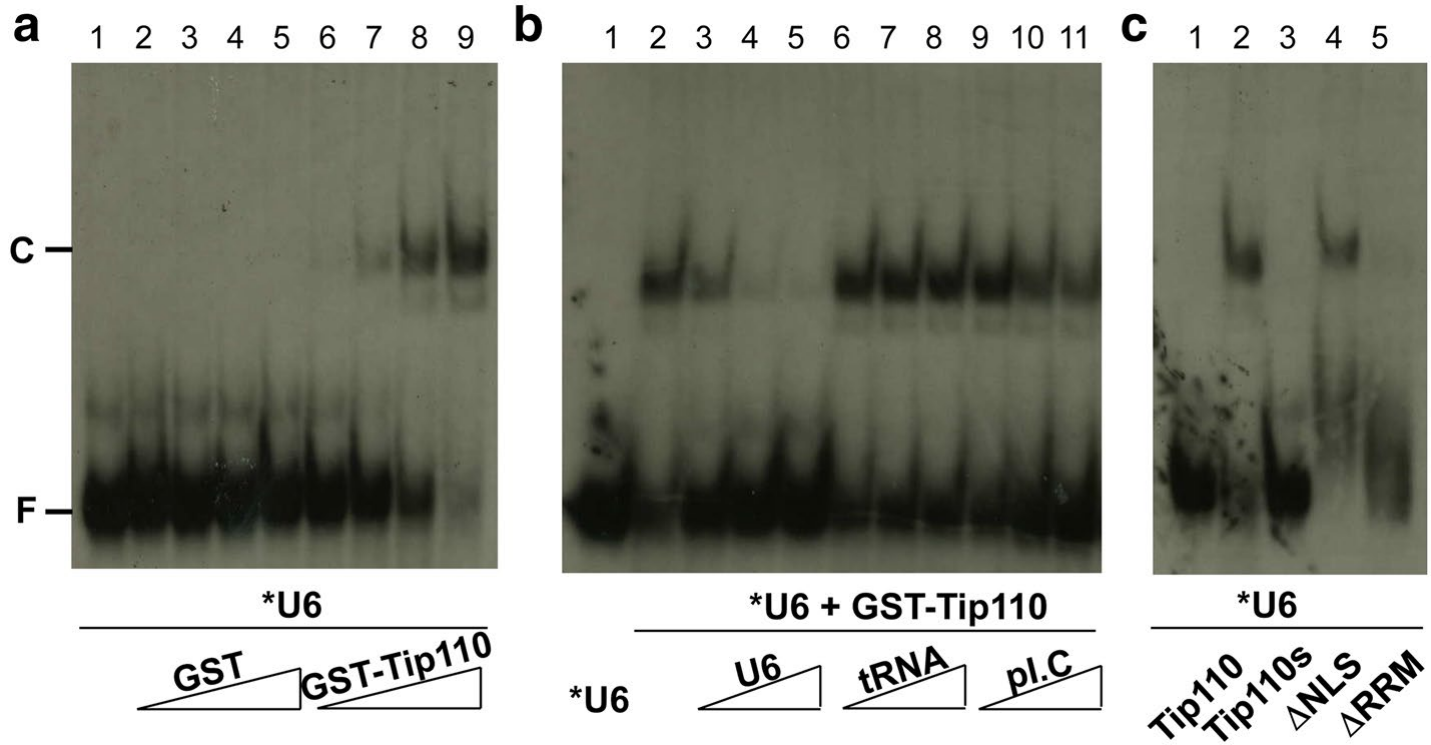
Specific binding of Tip110 protein to U6 RNA. **a** Complex formation between Tip110 and U6 RNA. ^32^P-labeled U6 RNA (*U6) was incubated with 1, 5, 10, and 100 ng recombinant GST (*lanes 2–5*) or GST-Tip110 (*lanes 6–9*). The reaction containing no recombinant protein was included to show free ^32^P-labeled U6 RNA (*lane 1*). The RNA–protein complex formation was determined using the electrophoretic mobility shift assay (EMSA). **b** Specificity of Tip110 binding to U6 RNA. ^32^P-labeled U6 RNA (*U6) was incubated with 10 ng recombinant GST-Tip110 in the presence of 5, 10, and 50 ng unlabeled U6 RNA (*lanes 3–5*), yeast tRNA (*lanes 6–8*), or polyinosinic:polycytidylic acid (poly I.C) (*lanes 9–11*). The complex formation of Tip110 and U6 RNA was determined using the EMSA. The binding reactions containing *U6 only (*lane 1*) and *U6 and GST-Tip110 (*lane 2*) were also included. **c** Binding of Tip110 mutants to U6 RNA. ^32^P-labeled U6 RNA (*U6) was incubated with 10 ng recombinant GST-Tip110s (*lane 3*), GST-ΔNLS (*lane 4*), or GST-ΔRRM (*lane 5*). The RNA–protein complex formation was determined using the EMSA. The binding reactions containing *U6 RNA only (*lane 1*) or *U6 RNA and GST-Tip110 (*lane 2*) were also included. *C* RNA–protein complex; *F* unbound free RNA.

To ascertain Tip110 binding to U6 RNA, we synthesized U6 RNA in vitro in the presence of α-^32^P-UTP and used the ^32^P-labeled U6 RNA as a probe. We then incubated 1, 5, 10 and 50 ng purified recombinant GST-Tip110 protein with ^32^P-labeled U6 RNA and determined Tip110-U6 complex formation using a gel mobility shift assay. The same amounts of purified GST protein were included as a control in the experiments. Complex formation between Tip110 and U6 became evident when 5 ng Tip110 was added and increased significantly when more Tip110 protein was present (Figure 2a). There was no complex formation between GST and U6 in all reactions. To determine the specificity of Tip110 binding to U6, we incubated 10 ng GST-Tip110 protein with ^32^P-labeled U6 RNA in the presence of 1-, 10-, and 100-fold excess amount of unlabeled U6 RNA, yeast tRNA and poly(I.C) and determined Tip110-U6 complex formation using the same assay. Increasing amounts of unlabeled U6 effectively competed out ^32^P-labeled U6 for Tip110 binding, as Tip110 and ^32^P-labeled U6 no longer formed a complex in the presence of unlabeled U6 RNA (Figure 2b). In contrast, inclusion of U6-unrelated yeast tRNA and poly(I.C) had little effects on the complex formation between Tip110 and ^32^P-labeled U6 RNA. There are two RRMs at the C-terminal of Tip110, one at aa700–778 and the other at aa802–874, and one nuclear localization signal (NLS) at aa601–650 [20]. To determine the role of the RRM domains in Tip110 binding to U6 RNA, we constructed, expressed and purified recombinant GST-Tip110 mutant that had both RRMs deleted (ΔRRM) and determined its ability to bind to U6RNA. Similarly, we also expressed, purified and included in these experiments recombinant Tip110 mutant ΔNLS containing no NLS and a naturally occurring small splicing variant that only encodes the N-terminal 365 amino acids of Tip110 (Tip110s) [20]. Compared to Tip110, both ΔRRM and Tip110s showed no binding to U6 RNA, while ΔNLS retained the U6 binding activity (Figure 2c), suggesting that RRMs were directly involved in Tip110 binding to U6 RNA. Collectively, these results confirm the finding from the yeast three-hybrid cloning that Tip110 binds to U6 RNA in a specific manner.

### Promotion of pre-mRNA splicing by Tip110

**Figure 3:**
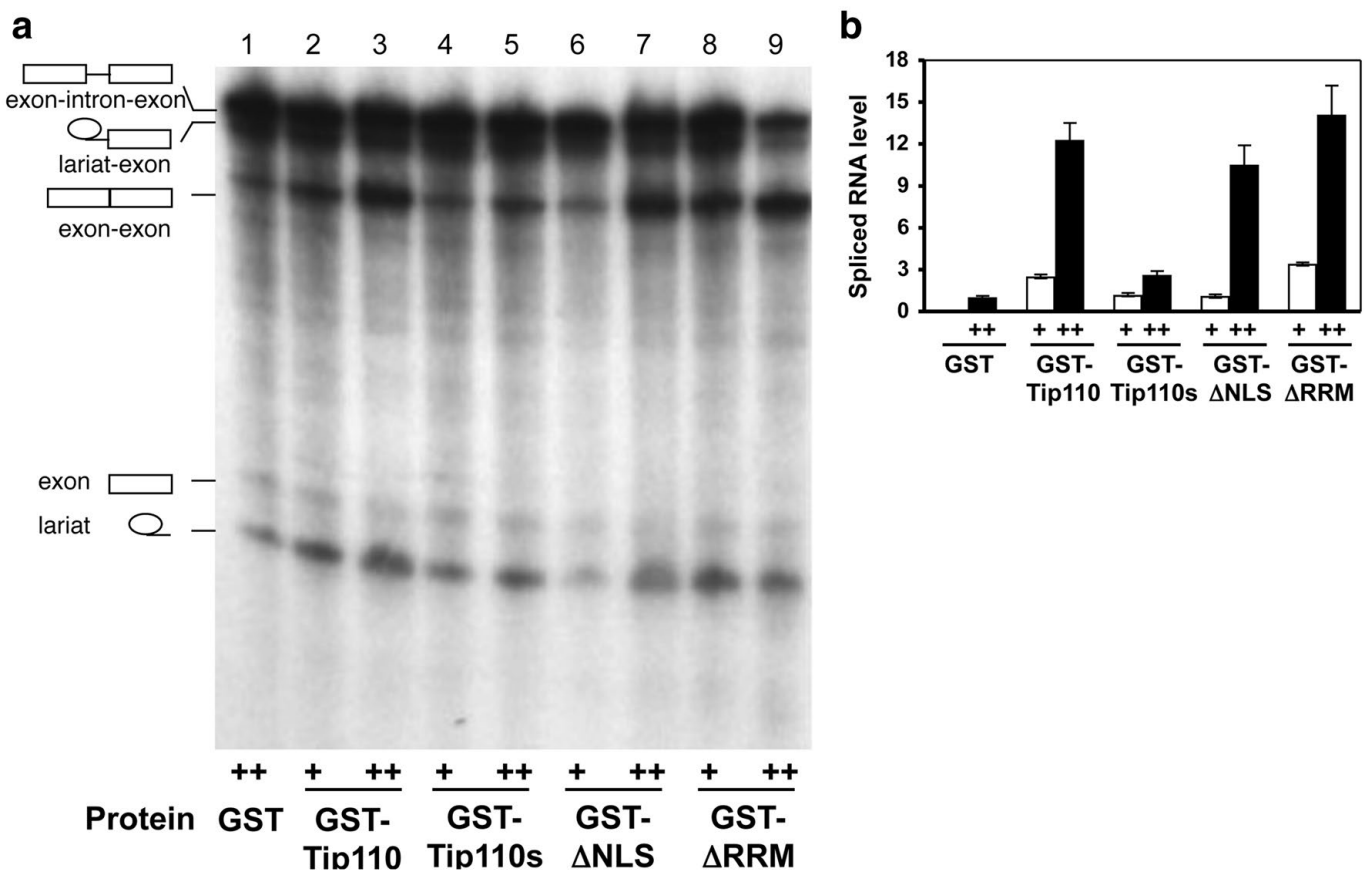
Human β-hemaglobin pre-mRNA splicing by Tip110 and its mutants. ^32^P-labeled human β-hemaglobin minigene pre-mRNA was incubated with Hela nuclear extract in the presence of 10 (+) or 50 ng (++) recombinant GST-Tip110 (*lanes 2 *and* 3*), GST-Tip110s (*lanes 4 *and* 5*), GST-ΔNLS (*lanes 6* and* 7*), or GSTΔRRM (*lanes 8* and* 9*). Recombinant GST protein (50 ng) was included as a control (*lane 1*). The reaction mixtures were separated on 8% denaturing PAGE (**a**). The *symbols on the left* are pre-mRNA and its splicing products. Splicing was quantitated by densitometric scanning of the intronless spliced RNA product on the autoradiography, the spliced RNA product in the reaction containing GST (*lane 1*) was set as the reference to calculate the relative level of pre-mRNA splicing (**b**).

Small nuclear U6 RNA directly participates in pre-mRNA splicing [35]. Our early studies have shown that Tip110 is localized in the spliceosome-rich “speckles” subnuclear structure [20]. Thus, the finding of Tip110 binding to U6 prompted us to determine the possible role of Tip110 in eukaryotic pre-mRNA splicing. We then performed an in vitro splicing assay using a human β-globin minigene pre-mRNA as a substrate. This minigene encodes the first exon, the first intron, and a major part of the second exon of human β-globin and has been well characterized and widely used in the in vitro splicing assay [36, 37]. We added 10 and 100 ng purified GST-Tip110 protein in the splicing reaction and determined its effects on the β-globin pre-mRNA splicing (Figure 3a). We also included 100 ng GST protein as a control. Compared to the GST control, 10 and 100 ng GST-Tip110 increased the splicing efficiency by about 3-fold and 12-fold, respectively (Figure 3b). We also added GST-Tip110s, GST-ΔNLS and GST-ΔRRM in the splicing reaction. GST-Tip110s only showed a very modest increase in splicing, while both GST-ΔNLS and GST-ΔRRM enhanced the pre-mRNA splicing similarly to that of the GST-Tip110 (Figure 3b). These results demonstrate that Tip110-mediated pre-mRNA splicing and that the domain responsible for this function was located at the C terminus of the protein. In addition, promotion of pre-mRNA splicing by GST-ΔRRM suggest that Tip110 might function in other steps of pre-mRNA splicing, at least in the context of the in vitro splicing assay.

### Inhibition of pre-mRNA splicing by Tip110 depletion and its reversal by Tip110 add-back

**Figure 4:**
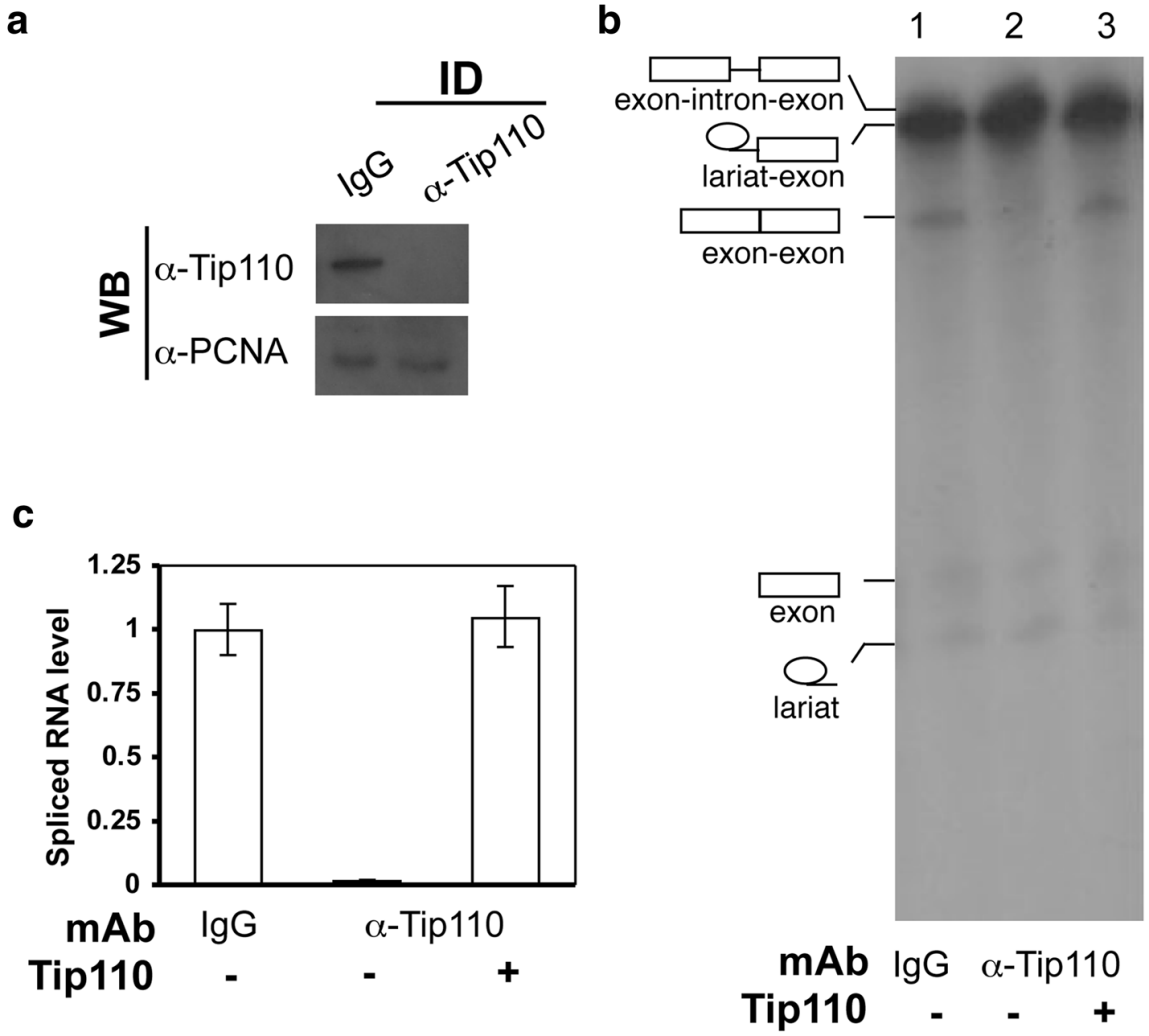
Effects of Tip110 immunodepletion and add-back on pre-mRNA splicing. Hela nuclear extract was pre-incubated with Tip110 mAb-conjugated protein-A beads to deplete endogenous Tip110 protein, as determined by Western blot analysis (**a**), and then used for pre-mRNA splicing reaction (**b**). An isotype-matched immunoglobulin control (IgG) was included as an immunodepletion control. Tip110-depleted nuclear extract was supplemented with recombinant Tip110 (100 ng) for the splicing reaction (**b**). Splicing was quantitated by densitometric scanning of the intronless spliced RNA product on the autoradiography, the spliced RNA product in the reaction containing the control IgG-immunodepleted Hela nuclear extract was set as the reference to calculate the relative level of pre-mRNA splicing (**c**).

To determine the significance of Tip110 function in pre-mRNA splicing, we decided to immunodeplete Tip110 protein from the Hela nuclear extract that was used in the in vitro splicing assay. Thus, we first generated anti-human Tip110 monoclonal antibodies using recombinant GST-Tip110. We then incubated Hela nuclear extract with anti-human Tip110 antibodies and protein-A agarose beads. Tip110-depleted Hela nuclear extract was recovered by brief centrifugation, followed by removal of the beads. An isotype-matched IgG was included as a control. Western blot analysis showed that immunodepletion efficiently removed Tip110 protein from Hela nuclear extract (Figure 4a). We used these extracts and performed the pre-mRNA splicing assay (Figure 4b). Compared to the IgG control, little splicing products were detected in anti-Tip110 antibody-depleted Hela nuclear extract (Figure 4c). To ascertain Tip110 function in pre-mRNA splicing, we performed the splicing assay by adding back GST-Tip110 protein into Tip110-depleted Hela nuclear extract. The results showed that Tip110 add-back restored the splicing activity. Taken together, these results show that Tip110 is indispensible for constitutive pre-mRNA splicing.

### Correlation between Tip110 expression and pre-mRNA splicing-dependent reporter gene expression

**Figure 5:**
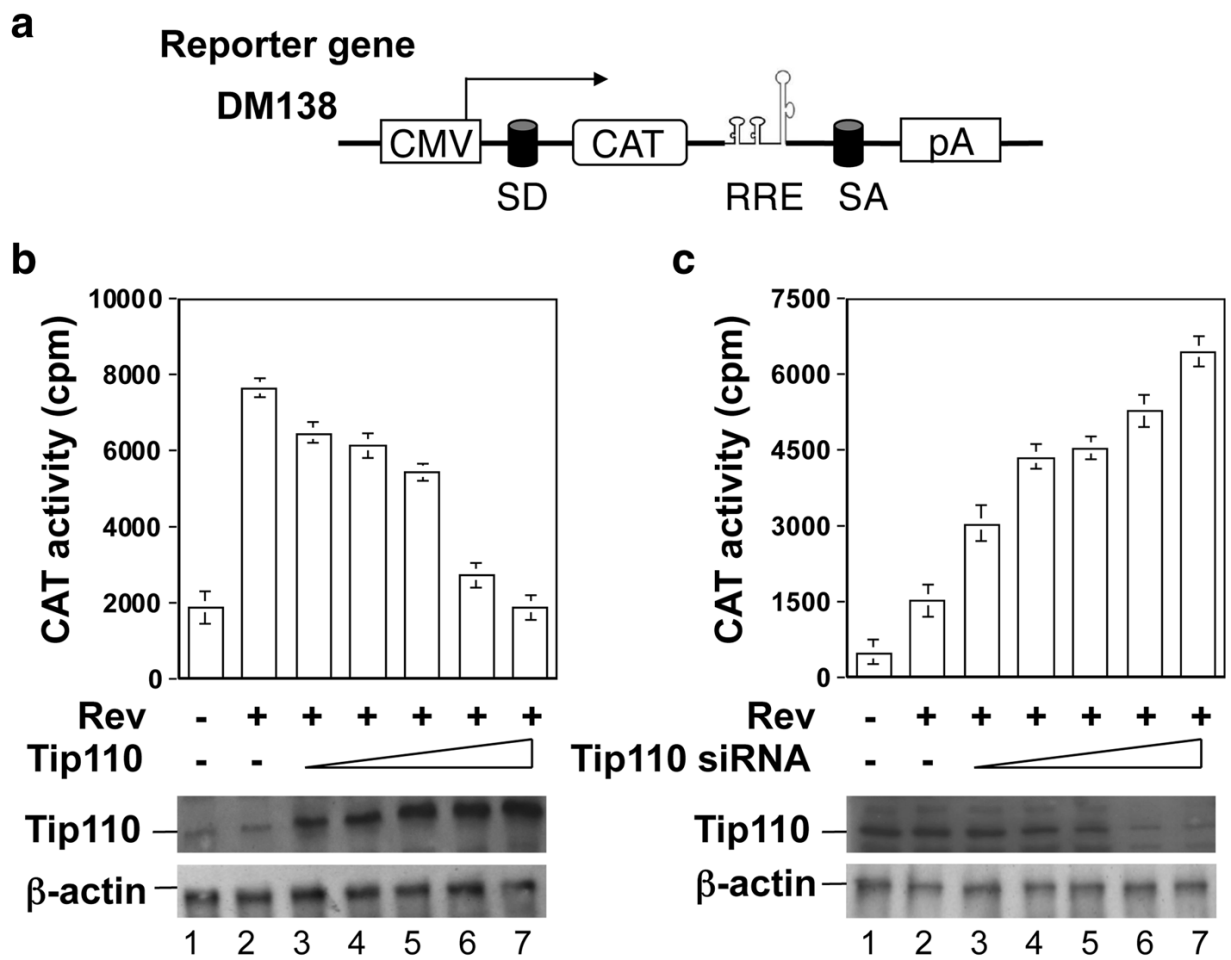
Effects of Tip110 expression on pre-mRNA splicing-dependent CAT reporter gene expression. A. Scheme of the HIV-1 Rev protein-dependent intron-containing CAT reporter DM138. the CAT gene expression requires nuclear export of intron-containing (unspliced) CAT transcript through the interaction between HIV-1 Rev protein and Rev responsive element (RRE) within the CAT transcript. *SD* splice donor, *SA* splice acceptor. **b** 293T cells were transfected with DM138 (*lane 1*), DM138 and Rev (*lane 2*), or DM138, Rev and increasing amounts of Tip110 (*lanes 3–7*). The cells were harvested 48 h after transfection for CAT assay (*top panel*) or Western blot for Tip110 (*bottom panel*). **c** 293T cells were transfected with DM138 (*lane 1*), DM138 and Rev (*lane 2*), or DM138, Rev and increasing amounts of Tip110 siRNA expression plasmids (*lanes 3–7*). The cells were harvested 48 h after transfection for CAT assay (*top panel*) or Western blot for Tip110 (*bottom panel*). The amount of total DNA in all transfections was equalized with pcDNA3 plasmid. A CMVβGal plasmid was included to normalize the transfection variations for CAT assay, while β-actin was included to show equal loading (**b**, **c**). Compared to Figure 5b, c only used one fifth of DM138 reporter plasmid in the transfections.

Although pre-mRNA splicing mainly occurs in the nucleus, some processes involve both cytosol and nucleus [38]. It is clear that the in vitro splicing assay is likely not representative some aspects of in vivo splicing process. Thus, we decided to further determine Tip110 function in pre-mRNA splicing in vivo. We took advantage of a HIV-1 Rev-dependent reporter gene DM138 (Figure 5a) and determined the relationship between Tip110 expression and pre-mRNA splicing. The chloramphenicol acetyltransferase (CAT) reporter gene in DM138 is under the control of the CMV promoter, is flanked by introns containing splice donor and acceptor sites, and has the CAT gene linked to a DNA sequence encoding HIV-1 Rev responsive element (RRE) [39, 40]. The construct is designed in such a manner that the CAT pre-mRNA transcripts contain the introns and RRE, which will not be exported into the cytosol from the nucleus for CAT protein translation unless HIV-1 Rev is provided *in trans*. We reasoned that if Tip110 enhanced pre-mRNA splicing, Tip110 expression would negate HIV-1 Rev-dependent expression of intron-containing pre-mRNA. Thus, we transfected 293T cells with DM138 alone, or in combination with HIV-1 Rev plasmid and increasing amount of Tip110 expression plasmid. We then determined CAT activity. As expected, Rev expression significantly improved CAT gene expression (Figure 5b). On the other hand, Tip110 expression decreased Rev-dependent CAT gene expression, and the decrease appeared to be dose-dependent. Furthermore, we also knocked down constitutive Tip110 expression using a Tip110 siRNA expression plasmid and determined its effects on CAT gene expression. In contrast to Tip110 over-expression, Tip110 knockdown increased CAT gene expression, and the increase was also dose-dependent (Figure 5c). The inverse correlation between Tip110 expression and pre-mRNA splicing dependent CAT reporter gene expression confirm that Tip110 is directly involved in eukaryotic pre-mRNA splicing.

### Requirement of Tip110 domains and pre-mRNA splicing

**Figure 6:**
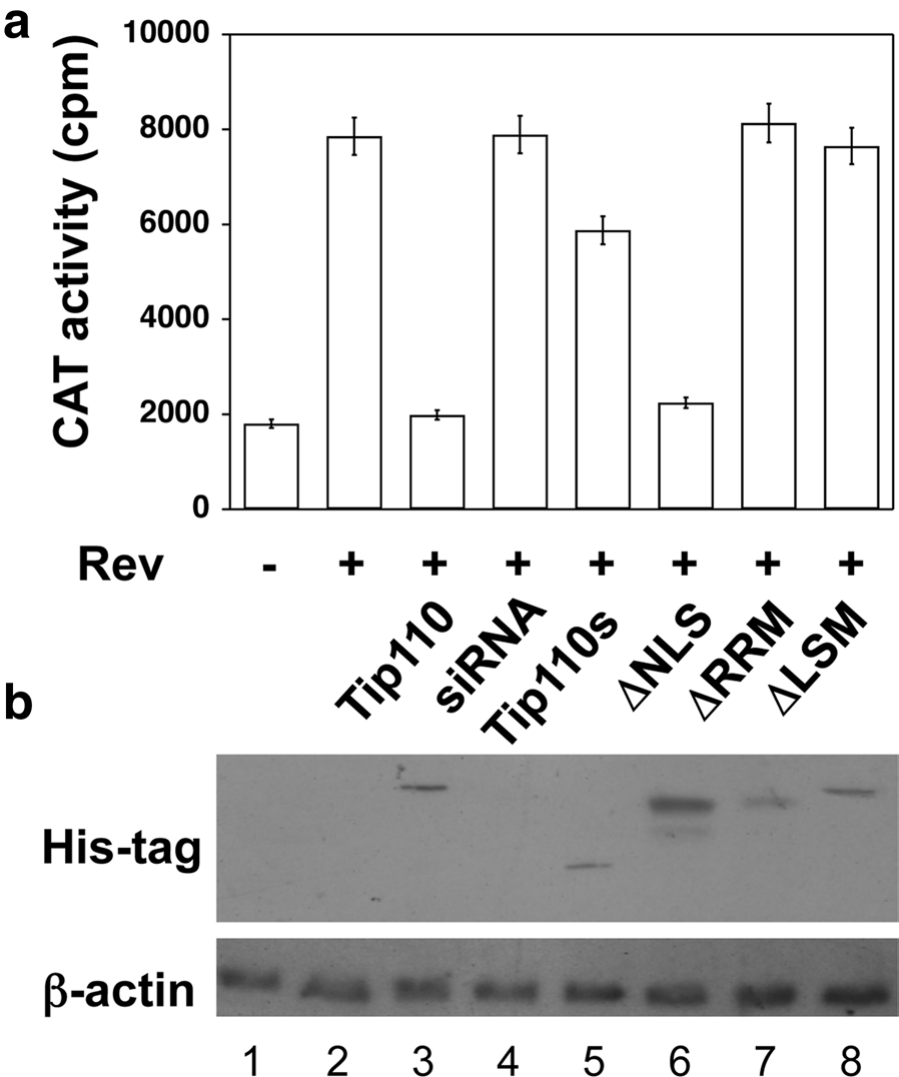
Effects of Tip110 mutants on pre-mRNA splicing-dependent CAT reporter gene expression. 293T cells were transfected with DM138 (*lane 1*), DM138 and Rev (*lane 2*), or DM138, Rev, and Tip110 (*lane 3*), Tip10 siRNA (*lane 4*), Tip110s (*lane 5*), ΔNLS (*lane 6*), ΔRRM (*lane 7*), or ΔLSM (*lane 8*). The cells were harvested 48 h after transfection for CAT assay (**a**) or Western blot for expression of Tip110 and its mutants (**b**). The amount of total DNA in all transfections was equalized with pcDNA3 plasmid. A CMVβGal plasmid was included to normalize the transfection variations for CAT assay, while β-actin was included to show equal loading.

Next, we performed the same reporter gene assay using different Tip110 deletion mutants and further examined the structure–function relationship of Tip110 in terms of pre-mRNA splicing. We transfected 293T cells with DM138 alone, or in combination with HIV-1 Rev plasmid, and Tip110s, ΔNLS, or ΔRRM expression plasmid. We also included full-length Tip110 and Tip110 siRNA expression plasmids in these experiments. In agreement with previous results (Figure 5), Tip110 over-expression decreased CAT gene expression (Figure 6a). As expected, Tip110 siRNA expression increased CAT gene expression. On the other hand, over-expression of Tip110 or ΔRRM showed no effects on CAT gene expression. Interestingly, over-expression of ΔNLS inhibited CAT gene expression, but to a lesser extent than that of full-length wild-type Tip110. In addition, we also constructed a mutant ΔLSM lacking the Lsm motif located between aa942–963, which has been postulated to interact with Lsm proteins to promote U4/U6 formation [41]. Similar to Tip110s and ΔRRM, ΔLSM over-expression had no effects on CAT gene expression. We also performed a Western blot analysis to ensure comparable protein expression of Tip110 and its mutants (Figure 6b). These results suggest that the U6-binding domain RRM, the Lsm proteins-interacting domain LSM, and NLS are all important for Tip110 function in pre-mRNA splicing.

### Presence of Tip110 in the splicing complexes

**Figure 7:**
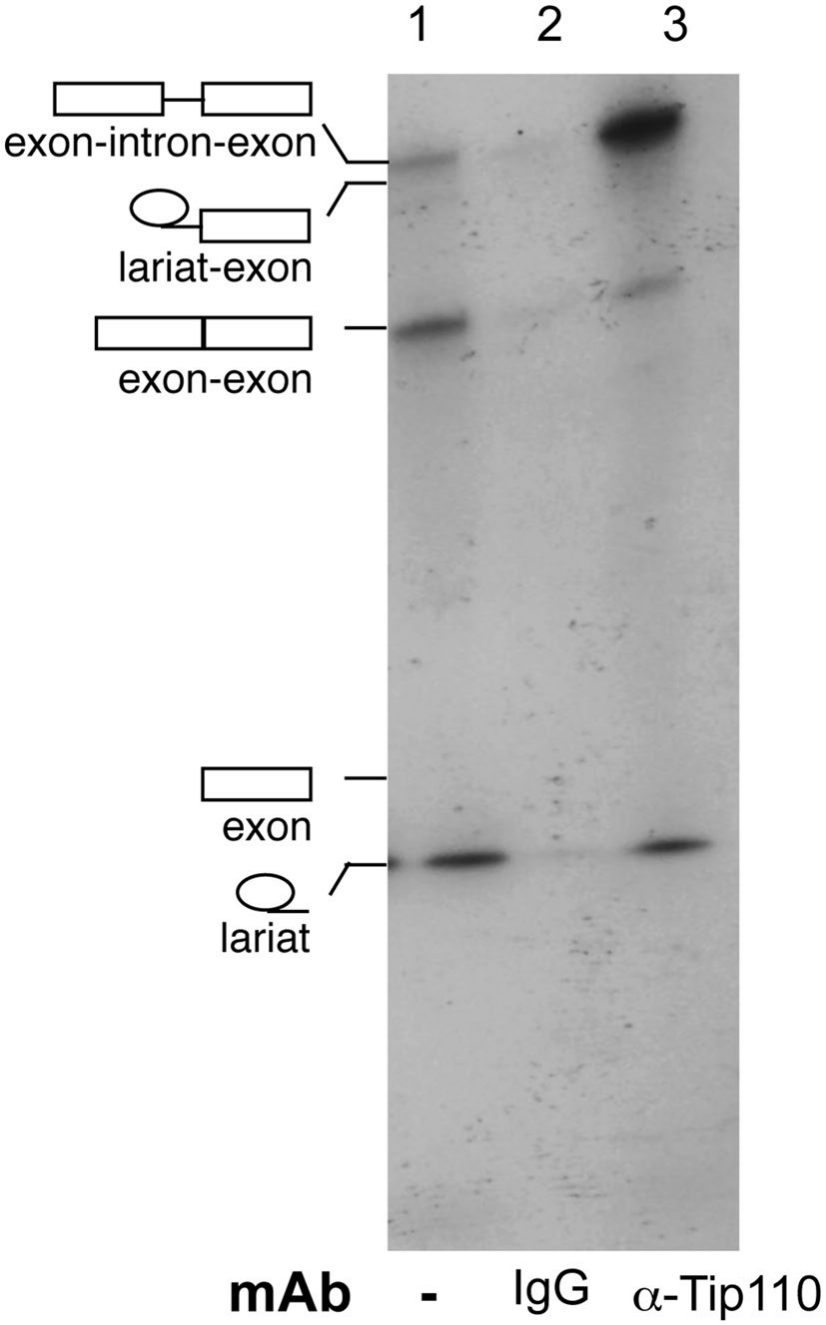
Tip110 association with pre-mRNA and spliced RNA products in the spliceosomes. ^32^P-labeled human β-hemaglobin minigene pre-mRNA was first incubated with Hela nuclear extract (*lane 1*). The splicing reaction was then immunoprecipitated with an anti-Tip110 monoclonal antibody (*lane 3*), or an isotype-mached IgG control (*lane 2*). The immunprecipitates were subjected to 8% denaturing PAGE. The *symbols on the left* are pre-mRNA and its splicing products.

To test whether Tip110 is physically associated with active spliceosomes, we incubated an in vitro splicing reaction with immobilized anti-Tip110 antibody, immunoprecipitated the labeled RNA species in the splicing reaction, and analyzed the immunoprecipitated RNA species. An isotype-matched IgG was included as a control. In addition, the splicing reaction without any IgG was also included as a mock control. Compared to the mock control, no significant RNA species were detected in the IgG control immunoprecipitates (lane 2, Figure 7), whereas more unspliced β-globin pre-mRNA and less intron-spliced β-globin mRNA were detected in the anti-Tip110 immunoprecipitates (lane 3, Figure 7). Of note was that released lariat RNA species was also detected in the anti-Tip110 immunoprecipitates. These results confirmed that Tip110 was associated with active spliceosomes and provide further support for Tip110 function in pre-mRNA splicing.

### Effects of Tip110 on global pre-mRNA splicing

**Figure 8:**
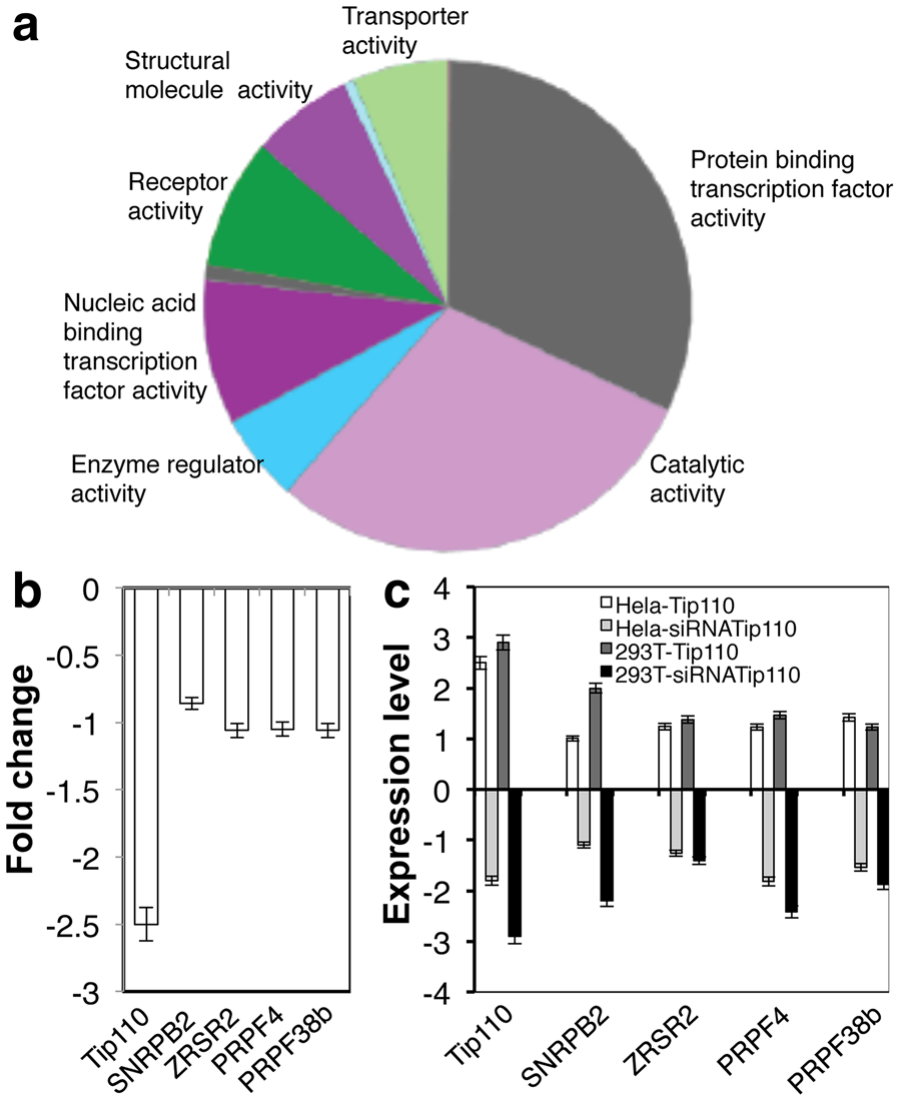
Tip110 global regulation on alternate pre-mRNA splicing and validation of Tip110-regulated four splicing factors. **a** Hela were transfected with Tip110-specific siRNA and the control siRNA. Cells were harvested for RNA isolation, the RNA was used for RNA-Seq. Panther pathway analysis was performed to group the total of 17,498 genes with changes in alternate pre-mRNA splicing. **b** Four splicing factors were down-regulated at the mRNA level from RNA-Seq. **c** qRT-PCR was performed to validate these four mRNA expression from Hela and 293T that were either transfected with Tip110 expression vector and siRNA for Tip110. cDNA3 was included as the control for Tip110; siRNA control was included as the control for Tip110 siRNA. The fold changes were calculated using the control as the reference (**b**, **c**).

We next determined whether Tip110 expression would globally affect pre-mRNA splicing. We transfected Hela with Tip110-specific siRNA or control siRNA, isolated mRNA and performed the next generation RNA sequencing (RNA-Seq). The cells transfected with Tip110 siRNA and control siRNA were compared for the alternative gene spliced transcripts. Bioinformatics analysis of the RNA transcripts indicated that Tip110 knockdown led to changes of alternative spliced RNA transcripts of 17,498 genes with statistical significant difference (*p* < 0.05). Using the Panther pathways analysis, those genes were mainly grouped as the following categories: protein-binding transcription factors (37%), catalytic molecules (35%), nucleic acid-binding transcription factors (9%), structural molecules (6%), and receptors (4%) (Figure 8a). Of particular note were four pre-mRNA splicing factors snRNP polypeptide B (SNRPB2), zinc finger RNA-binding motif and serine/arginine-rich 2 (ZRSR2), pre-mRNA processing factor 4 (PRPF4) and pre-mRNA processing factor 38b (PRPF38b) that showed all down-regulation in Tip110 siRNA-transfected cells (Figure 8b). qRT-PCR was then performed to quantify the mRNA expression of these four genes in Tip110 siRNA-transfected Hela as well as in Tip110-transfected Hela. The results showed the positive association between Tip110 and mRNA levels of these four genes (Figure 8c). Similar results were obtained in 293T. Taken together, these results showed that Tip110 exhibited global effects on expression of host genes including the pre-mRNA splicing factors and suggest that Tip110-induced changes in pre-mRNA splicing factors may provide additional regulatory control of Tip110-mediated pre-mRNA splicing.

## Discussion

In this study we aimed to identify the RNA targets for Tip110 binding. We demonstrated that Tip110 specifically interacted with small nuclear U6 RNA through the yeast 3-hybrid screen and RNA EMSA (Figures 1, 2). Tip110 promoted pre-mRNA splicing in vitro (Figure 3) and in a pre-mRNA splicing-dependent reporter gene assay (Figure 5). Corroborated with these findings were that depletion of Tip110 from nuclear extract led to inhibition of pre-mRNA splicing and that add-back Tip110 restored pre-mRNA splicing (Figure 4). Moreover, RRM domain of Tip110 was shown to be important for U6 RNA binding; RRM, LSM, and NLS domains were all important for Tip110 function in pre-mRNA splicing (Figures 2, 6). Furthermore, Tip110 was detected in association with pre-mRNA- and spliced RNA-containing spliceosomes (Figure 7). Lastly, RNA-Seq analysis showed that Tip110 expression was linked to alternate pre-mRNA splicing of the majority of genes in human genome including several splicing factors (Figure 8). These results demonstrated that Tip110 regulated global pre-mRNA splicing likely through interaction with snRNA U6 and other splicing factors.

Biogenesis of the snRNP U1, U2, U4, U5, and U6 is a complex process. Newly synthesized snRNP undergo various modification and assembly, and then export from nuclear to cytoplasm where they mature and transport back into the Cajal bodies in the nucleus. snRNA/snRNP then recruit other splicing factors, undergo further assembly and maturation in the Cajal bodies [42–44]. Our current study identified U6 snRNA to be the putative Tip110-binding target through the yeast three hybrid cloning (Figure 1) and further confirmed the binding specificity through the gel shift mobility assay (Figure 2). These results are consistent with the previous reports that the U6 snRNP is targeted to the CB by specific binding to Tip110, followed by Tip110-induced assembly of U4/U6 snRNP for recycling of U4 and U6 snRNPs [27, 28, 41, 45]. Tip110 has been shown to localize not only in Cajal bodies, and is also required for the induction of CB formation as well [14, 31, 46]. These studies together suggest that Tip110 not only targets U6 snRNP to Cajal bodies through direct interaction, but also is required for snRNP U6 assembly into mature U4/U6.U5 tri-snRNP to exit Cajal bodies. When we further defined the Tip110 regions that were responsible for the snRNA U6 binding, we made a series of deletion mutants which including the C-terminal (1–350aa), deletion of NSL (∆600–670aa), deletion of two RRM (∆740–850aa). The C-terminal truncated protein had no binding activity at all, the deletion of NSL didn’t affect the binding, but deletion of the two RRM could abort the binding activity to about 80% (Figures 2, 6). Thus, the two RRM domains are very important to maintain the U6 binding activity of Tip110.

After exiting Cajal bodies, mature snRNP enter nuclear speckles. Nuclear speckles are sites for pre-mRNA splicing that serve as storage and/or modification sites for splicing factors [47]. Our previous digital fluorescence microscopic imaging revealed that Tip110 was detected within the nuclear speckle structure [20]. Tip110 may shuttle from Cajal bodies to nuclear speckles, likely along with the mature spliceosomes for pre-mRNA splicing, as both Cajal bodies and nuclear speckles are enriched in the snRNP U1, U2, U4, U5, and U6 [48]. Tip110 was shown to bind to RNPS1 at its C terminus and could be involved in constitutive and alternative pre-mRNA splicing [16]. RNPS1 is expressed in nuclear speckles and found in the active spliceosomes and functions as a general activator of pre-mRNA splicing [49, 50]. Our current study showed that Tip110 played important functions in pre-mRNA splicing (Figures 3, 4, 5) and was present in the spliceosomes (Figure 7). Therefore, it is likely that Tip110 interaction with RNPS1 and co-localization in nuclear speckles structure are also important for Tip110 function in pre-mRNA splicing. Transcription and pre-mRNA splicing are believed to be coupled by the C-terminal domain (CTD) of the large subunit of RNA polymerase II (Pol II) [1]. CTD phosphorylation is required for the stimulatory effect of the CTD on splicing [51, 52]. We have previously shown that Tip110 bound to the C-terminal domain of unphosphorylated RNA polymerase II in a direct and specific manner and Tip110 expression was associated with increased phosphorylation of serine 2 of the heptapeptide repeats within the RNAPII C-terminal domain [21]. Thus, Tip110 association with unphosphorylated RNAPII CTD provides another line of evidence to support direct involvement and function of Tip110 in pre-mRNA splicing.

To further determine the significance of Tip110 involvement in pre-mRNA splicing, we performed RNA-Seq analysis of the alternate pre-mRNA splicing transcripts. As expected, Tip110 knockdown led to alterations in a large number of the gene transcripts including four known genes in pre-mRNA splicing (Figure 8). Tip110 knockdown significantly decreased expression of PRPF4, PRPF38b, SNRPB2, and ZRSR2. PRPF4 is a component of the U4/U6 di-snRNP and the U4/U6.U5 tri-snRNP and is important for maintaining the pre-mRNA splicing efficiency [53]. SNRPB2 is also important for maintaining the pre-mRNA splicing efficiency [54]. ZRSR2 is a splicing factor engaged in the initial steps of pre-mRNA splicing, including 3′ splice-site recognition [55]. PRPF38b interacts (directly or indirectly) with at least one of the U6 snRNA-containing snRNP complexes and is required for pre-mRNA splicing [56]. These findings suggest that Tip110 regulation of these key pre-mRNA splicing factors, is, if not more, as important as its interaction with snRNA U6 in pre-mRNA splicing. Tip110 is barely detectable in normal tissue and cells and highly elevated in tumors and differentiating stem cells [20, 25]. Thus, it is possible that Tip110 function in pre-mRNA splicing is evident only in cancerous cells and tumors through which elevated Tip110 expression causes changes in pre-mRNA splicing and gene expression and subsequent stem cell differentiation and malignant transformation.
